# Comorbidities of Pediatric Patients With Hidradenitis Suppurativa in the Emergency Department Setting: A Cross‐Sectional Study

**DOI:** 10.1111/pde.70054

**Published:** 2025-10-13

**Authors:** Rahwa Hailemichael, Sydney A. Martin, Austin Hwang, Julia M. Riley, Kelsey S. Flood, Ziyou Ren

**Affiliations:** ^1^ Department of Dermatology Feinberg School of Medicine, Northwestern University Chicago Illinois USA

**Keywords:** comorbidities, emergency department, hidradenitis suppurativa, HS, pediatric dermatology

## Abstract

Hidradenitis suppurativa (HS) often leads to painful episodes requiring emergency department (ED) care, yet little is known about the comorbidities associated with these visits in pediatric patients. This study examined the Nationwide Emergency Department Sample (NEDS) from 2006 to 2019, identifying HS diagnoses via ICD‐9 and ICD‐10 codes to evaluate associated comorbidities. Pediatric HS ED visits were linked with 17 comorbidities, including systemic lupus erythematosus, as well as various psychosocial, endocrine, and dermatologic conditions. These findings highlight the need for expanded comorbidity screening in pediatric HS care.

## 1 Introduction

Hidradenitis suppurativa (HS) is a chronic inflammatory skin condition caused by follicular occlusion, leading to painful nodules, sinus tracts, and scarring. HS is associated with metabolic syndrome, depression, inflammatory bowel disease, and inflammatory arthritis in adults, but pediatric studies are limited [1, 2]. This study aimed to identify comorbidities in pediatric patients with HS utilizing emergency department (ED) services.

## 2 Methods

This study was approved by Northwestern IRB STU00214408. Data were integrated from 2006 to 2019 Nationwide Emergency Department Sample (NEDS), providing an annual 20% stratified cross‐sectional sample of US hospital EDs. All data were de‐identified and limited to pediatric patients (< 18 years old). Diagnosis of HS was identified using ICD‐9705.83 and ICD‐10 L73.2 codes. Identification of HS and comorbidities is presented in Data S1. Statistical analysis was conducted using SAS 9.4. Descriptive statistics, including weighted means and confidence intervals, were calculated, and a chi‐square test was performed to determine the *p* value. Odds ratio was adjusted for age, gender, and insurance status via logistic regression model.

## 3 Results

Pediatric patients with HS in the ED had increased prevalence of anxiety and depression compared to patients without HS but showed decreased risk due to strong confounding effects. No significant association was observed for tobacco use, schizophrenia, opioid use, or homelessness. HS was associated with a higher prevalence of psoriasis (OR = 4.68), acne (OR = 10.44), pilonidal cyst (OR = 4.59), pyoderma gangrenosum (OR = 52.64) (all *p* < 0.0001), and atopic dermatitis (OR = 2.07; *p* = 0.04) (Table 1). HS was not statistically associated with vitiligo.

**TABLE 1:** Frequency and odds ratios for comorbidities and social determinants of health of HS using ICD‐9 and ICD‐10 coding.

Primary diagnosis of disease	Children with HS (weighted *n* = 31,362)		Children without HS (weighted *n* = 404,000,000)		Univariate model odds ratios [95% CI]	*p*	Multivariate model^a^ odds ratios [95% CI]	*p*
	Weighted frequency (95% CI)	Weighted % prevalence (95% CI)	Weighted frequency (95% CI)	Weighted % prevalence (95% CI)				
Conditions of skin and soft tissue								
Psoriasis	49	0.1 (0.02–0.18)	46,848	0.01 (0.0112–0.0120)	8.66 [3.79–19.75]	**< 0.0001**	4.68 [2.05–10.67]	**< 0.0001**
Acne	365	0.75 (0.56–0.94)	203,731	0.05 (0.0485–0.0522)	15.09 [11.71–19.45]	**< 0.0001**	10.44 [8.07–13.51]	**< 0.0001**
Pyoderma gangrenosum	13	0.027 (0–0.06)	882	0.0002 (0.0002–0.0003)	124.94 [39.61–394.06]	**< 0.0001**	52.64 [16.30–168.93]	**< 0.0001**
Pilonidal cyst	417	0.86 (0.67–1.05)	240,379	0.06 (0.0581–0.0608)	14.54 [11.64–18.17]	**< 0.0001**	4.59 [3.67–5.75]	**< 0.0001**
Vitiligo	10	0.02 (0–0.05)	19,694	0.0049 (0.0045–0.0053)	4.19 [1.05–16.78]	**0.043**	2.54 [0.64–10.15]	**0.19**
Atopic dermatitis	52	0.11 (0.03–0.18)	496,022	0.12 (0.11–0.13)	0.87 [0.44–1.72]	0.69	2.07 [1.04–4.10]	**0.04**
Psychosocial								
Depression	850	1.75 (1.37–2.12)	4,141,559	1.02 (0.99–1.06)	1.74 [1.41–2.16]	< 0.0001	0.54 [0.44–0.67]	**< 0.0001**
Anxiety	640	1.32 (1.05–1.59)	3,304,859	0.8396 (0.8121–0.8671)	1.58 [1.29–1.94]	< 0.0001	0.51 [0.41–0.62]	**< 0.0001**
Tobacco use	1464	3.01 (2.60–3.42)	4,153,895	1.03 (0.97–1.08)	2.99 [2.64–3.39]	< 0.0001	0.89 [0.78–1.01]	0.07
Schizophrenia	68	0.14 (0.07–0.21)	316,233	0.08 (0.0751–0.0813)	1.80 [1.09–2.97]	0.02	0.67 [0.41–1.11]	0.12
Opioid use	20	0.04 (0.0005–0.0820)	120,889	0.03 (0.0283–0.0315)	1.38 [0.52–3.70]	0.52	0.47 [0.18–1.26]	0.13
Homelessness	6	0.01 (0–0.04)	34,788	0.0086 (0.0078–0.0094)	1.40 [0.20–9.95]	0.73	0.68 [0.10–4.81]	0.7
Endocrine								
Metabolic syndrome	112	0.23 (0.13–0.34)	19,073	0.0047 (0.0042–0.0052)	49.23 [31.44–77.11]	< 0.0001	17.98 [11.47–28.18]	**< 0.0001**
Diabetes mellitus type 1	416	0.85 (0.67–1.04)	866,842	0.21 (0.20–0.22)	4.01 [3.23–5.00]	< 0.0001	1.83 [1.47–2.28]	**< 0.0001**
Diabetes mellitus type 2	1058	2.18 (1.80–2.55)	842,336	0.21 (0.20–0.21)	10.69 [8.98–12.74]	< 0.0001	NA	NA
Obesity	2661	5.47 (4.81–6.14)	1,019,153	0.25 (0.24–0.26)	22.97 [20.37–25.90]	< 0.0001	8.90 [7.92–10.00]	**< 0.0001**
High cholesterol and lipid disorders	180	0.37 (0.35–0.69)	113,638	0.03 (0.0267–0.0295)	13.25 [8.55–20.53]	< 0.0001	5.24 [3.39–8.10]	**< 0.0001**
Polycystic ovary syndrome	249	0.51 (0.33–0.69)	59,000	0.015 (0.0139–0.0153)	35.34 [25.03–49.90]	< 0.0001	8.21 [5.82–11.57]	**< 0.0001**
Pulmonary								
Obstructive sleep apnea	253	0.52 (0.35–0.69)	221,242	0.05 (0.05–0.06)	9.55 [6.96–13.11]	< 0.0001	9.33 [6.86–12.71]	**< 0.0001**
Asthma	3420	7.03 (6.33–7.73)	27,082,437	6.7 (6.54–6.86)	1.06 [0.96–1.16]	0.28	0.96 [0.88–1.06]	0.43
Gastrointestinal								
Crohn's disease	248	0.51 (0.39–0.71)	161,646	0.04 (0.0375–0.0424)	12.84 [8.69–18.98]	< 0.0001	5.04 [3.43–7.43]	**< 0.0001**
Ulcerative colitis	20	0.04 (0–0.08)	61,273	0.02 (0.0140–0.0163)	2.69 [0.96–7.51]	0.06	1.06 [0.37–3.00]	0.92
Rheumatologic								
Systemic lupus erythematosus	105	0.22 (0.07–0.36)	81,983	0.02 (0.0375–0.0424)	10.80 [5.58–20.90]	< 0.0001	2.95 [1.53–5.72]	**0.0013**
Rheumatoid arthritis	67	0.14 (0.06–0.21)	93,465	0.02 (0.0221–0.0242)	6.00 [3.45–10.45]	< 0.0001	2.38 [1.37–4.16]	**0.0023**
Genetic								
Down syndrome	550	1.13 (0.06–0.21)	40,887	0.1 (0.10–0.11)	11.35 [8.81–14.63]	< 0.0001	20.40 [15.74–26.28]	**< 0.0001**

*Note:* Bold indicates statistically significant *p* values.

^a^ Adjusted for age, gender, and insurance status.

Metabolic syndrome (OR = 17.98), diabetes mellitus type 1 (OR = 1.83), high cholesterol and lipid disorders (OR = 5.24), obesity (OR = 8.90), polycystic ovarian syndrome (PCOS) (OR = 8.21), and obstructive sleep apnea (OSA) (OR = 9.33) were strongly associated with HS (all *p* < 0.0001).

Systemic lupus erythematosus (SLE) (OR = 2.95; *p* = 0.0013) and rheumatoid arthritis (OR = 2.38; *p* = 0.0023) were more prevalent in pediatric patients with HS. Down syndrome was 20.40 times more prevalent within our study cohort (*p* < 0.0001). Crohn's disease was significantly associated with HS (OR = 5.04; *p* < 0.0001), while ulcerative colitis was not (Table 1).

## 4 Discussion

Depression and anxiety are common in patients with HS, with pediatric rates reported at 19% and 22% [3], respectively. Similarly, our study demonstrated higher levels of depression and anxiety in pediatric HS patients utilizing the ED. However, after adjustment for age, gender, and insurance status, the association showed ORs less than 1, which indicates risks for depression and anxiety were actually lower in subgroups of HS patients. Therefore, screening for psychosocial comorbidities needs to consider patients' demographic and socioeconomic status in HS care.

Consistent with prior pediatric HS studies [3, 4], we observed a higher prevalence of acne and pyoderma gangrenosum. The association between atopic dermatitis and HS remains unclear [5, 6]. We found that pediatric patients with HS have 2.07 times greater odds of atopic dermatitis compared to those without. Given that 60% of patients develop atopic dermatitis in the first year of life [7], it may be a key comorbidity in pediatric HS. Similar to prior studies [3, 4], we found significantly increased odds of pilonidal cyst in pediatric patients with HS. Notably, pilonidal cyst was analyzed as a distinct comorbidity.

Our study also highlights the high burden of metabolic comorbidities in pediatric patients with HS. Metabolic syndrome, obesity, dyslipidemia, and insulin resistance were all correlated with HS in the pediatric ER setting. Psoriasis, PCOS, and OSA, all associated with metabolic comorbidities, were also more prevalent in our cohort. These findings suggest metabolic dysregulation may drive HS development. Future studies should evaluate whether poor comorbidity control exacerbates HS.

Limited reports have demonstrated an association between SLE and HS in adults [8, 9]. Our study found SLE to be significantly associated with pediatric HS. Adalimumab, a TNF‐alpha inhibitor approved for patients 12 years and older in 2018, has a well‐known side effect of drug‐induced lupus. Given this potential adverse reaction and their inflammatory etiologies, further exploration of this relationship is essential to guide therapeutic interventions.

Our study is limited by its cross‐sectional design and by its sole focus on patients presenting to the ED, which introduces bias toward individuals with more severe disease. Additionally, pediatric HS may present subtly, which may contribute to underdiagnosis and underrepresentation in ICD‐coded data.

## 5 Conclusion

This study highlights the complex nature of pediatric HS in the ED setting. A holistic approach to comorbidity screening is essential for comprehensive management and improving outcomes in pediatric patients with HS.

## Ethics Statement

This work was approved by Northwestern IRB; approval #STU00214408.

## Conflicts of Interest

Dr. Riley has served as an advisory board member for Novartis Pharmaceuticals. Dr. Flood has received fellowship funding from AbbVie, Janssen, and the National Psoriasis Foundation, paid directly to her institution. The other authors declare no conflicts of interest.

## Supporting information


**Data S1:** Supporting Information.

## Data Availability

The data that support the findings of this study are available from the corresponding author upon reasonable request.
