# Supplementary material for: Comorbidities of Pediatric Patients With Hidradenitis Suppurativa in the Emergency Department Setting: A Cross‐Sectional Study
Source: Pediatr Dermatol. 2025 Oct 13;43(2):365–8. doi: 10.1111/pde.70054 (PMC13051001; doi:10.1111/pde.70054)
Supplement: Supplementary file 1 — Data S1: Supporting Information. [file PDE-43-365-s001.docx]

**Supplemental Methods**

**Identification of Hidradenitis Suppurativa (HS) and comorbidities.**

NEDS lists one primary diagnosis, i.e., the condition chiefly responsible for the ED visit, and up to 29 secondary diagnoses using International Classification of Diseases (ICD)-code. ICD-9 was used from January 2006-September 2015. ICD-10 was used from October 2015. The databases were searched for a primary and/or secondary diagnosis of HS using ICD-9-CM code 705.83. For data after 2015, ICD-10 code of L73.2 was used to identify HS. The control group included all ED visits without a diagnosis of HS. Comorbidities were identified using ICD-9-CM, ICD-10 or Clinical Classification Software codes (Supplemental Table 1).

| **Supplemental Table 1. Codes used for identification of comorbidities.** | |  | |
| --- | --- | --- | --- |
| **Disease** | **ICD-9** | **ICD-10** | |
| Psoriasis | 6961, 6968 | L40.0, L40.1, L40.3, L40.4, L40.8, L40.9 | |
| Acne | 7061 | L70.0, L70.1, L70.2, L70.3, L70.4, L70.5, L70.9, L70.9, L73.0 | |
| Pyoderma Gangrenosum | 68601 | L88 | |
| Pilonidal Cyst | 6850, 6851 | L05.91, L05.01 | |
| Vitiligo | 70901 | L80, H02.731, H02.732, H02.734, H02.735, H02.736, H02.739 | |
| Eczema | 6918, 37331 | L20.82, L20.83, L20.84, L20.89, L20.9, L30.1 | |
| Depression | 29620, 2962, 29622, 29623, 29624, 29625, 29626, 29630, 29631, 29632, 29633, 29634, 29635, 29636 | | F31.4, F31.5, F31.9, F32.A, F32.2, F32.3, F32.89, F32.9,  F33.2, F33.3, F33.8, F33.9, F34.1, F41.2, F53.0, |
| Anxiety | 30000, 3000, 30002, 30009 | F06.4, F10.180, F10.280, F10.980, F13.180, F13.280, F15.180, F15.280, F40.10, F40.11, F41.0, F41.1, F41.8, F41.9, F45.21, F93.0 | |
| Tobacco Use | 3051, V1582, 98984, 64900, 64901, 64902, 64903, 64904 | F17.200, F17.201, F17.211, F17.221, F17.291, O99.33, O99.334, O99.335, P04.2, Z71.6, Z72.0, Z87.891 | |
| Schizophrenia | 29500, 29501, 29502, 29503, 29504, 29505, 29510, 29511, 29512, 29513, 29514, 29515, 29520, 29521, 29522, 29523, 29524, 29525, 29530,29531, 29532, 29533, 29534, 29535, 29540, 29541, 29542, 29543, 29544, 29545, 29550, 29551, 29552, 29553, 29554, 29555, 29560, 29561, 29562, 29563, 29564, 29565, 29570, 29571, 29572, 29573, 29574, 29575, 29580, 29581, 29582, 29583, 29584, 29585, 29590, 29591, 29592, 29593, 29594, 29595, V110 | F20.0, F20.2, F20.3, F20.5, F20.89, F20.9, F21, F21.1, F23, F25.0, F28, F29 | |
| Opioid Use | 30550 30551 30552, 30553, 30400, 30401, 30402, 30403, 30470, 3047, 30472, 30473 | F11.10, F11.11, F11.120, F11.121, F11.122, F11.129, F11.13, F11.14, F11.150, F11.151, F11.159, F11.181, F11.182, F11.19, F11.21, F11.221, F11.222, F11.229, F11.24, F11.281, F11.288, F11.90, F11.91, F11.92, F11.921, F11.922, F11.929, F11.93, F11.94, F11.950, F11.951, F11.959, F11.981, F11.982, F11.988, F11.99 | |
| Homelessness | V600, V601 | Z59.00, Z59.01, Z59.02, Z59.811, Z59.812, Z59.819 | |
| Metabolic Syndrome | 2777 | E88.81 | |
| Diabetes Mellitus Type 1 | 25001,25003, 25011, 25013, 25021, 25023, 25031, 25033, 25041, 25043, 25051, 25053, 25061, 25063, 25071, 25073, 25081, 25083, 25091, 25093 | E10.9, E10.10, E10.11, E10.36, E10.43, E10.44, E10.610, E10.618, O24.02, O24.92 | |
| Diabetes Mellitus Type 2 | 25000, 25002, 25010, 25012, 25020, 25022, 25030, 25032, 25040, 25042, 25050, 25052, 25060, 25062, 25070, 25072, 25080, 25082, 25090, 25092 | E11.00, E11.01, E11.1, E11.10, E11.11, E11.21, E11.22, E11.29, E11.319, E11.321, E11.329, E11.33, E11.331, E11.34, E11.341, E11.349  E11.35, E11.351, E11.353, E11.354, E11.355, E11.36, E11.37, E11.39, E11.41, E11.42, E11.43, E11.44, E11.49, E11.51, E11.52, E11.59, E11.610, E11.618, E11.620, E11.621, E11.628, E11.629, E11.630, E11.638, E11.641, E11.649, E11.65, E11.69, O24.12 | |
| Obesity | 27800, 27801, 64910, 64911, 64912, 64913, 64914, V8530, V8531, V8532, V8533, V8534, V8535, V8536, V8537, V8538, V8539, V8541, V8542, V8543, V8544, V8545 | E66.01, E66.09, E66.1, E66.2, E66.8, E66.9, O99.21, O99.214, O99.215 | |
| High Cholesterol & Lipid Disorders | 2720, 2721, 2723, 2722, 2624 | E78.00, E78.1, E78.2, E78.3, E78.49, E78.5 | |
| Polycystic Ovarian Syndrome | 2564 | E28.2 | |
| Obstructive Sleep Apnea | 32723 | G47.33 | |
| Asthma | 49300, 49301, 49302, 49310, 49311, 49312, 49390, 49391, 49392, 49320, 49321, 49322 | J45.20, J45.21, J45.22, J45.30, J45.31, J45.32, J45.40, J45.41, J45.42, J45.50, J45.51, J45.52, J45.901, J45.902, J45.909 | |
| Crohn’s Disease | 5550, 5551, 5552, 5559 | K50.00, K50.010, K50.011, K50.012, K50.013, K50.014, K50.019, K50.10, K50.111, K50.112, K50.113, K50.114, K50.118, K50.80, K50.811, K50.812, K50.813, K50.814, K50.819, K50.90, K50.911, K50.912, K50.913, K50.914, K50.918, K50.919 | |
| Ulcerative Colitis | 5560, 5561, 5562, 5563, 5565, 5566, 5568, 5569 | K51.80, K51.812, K51.813, K51.814, K51.818, K51.819, K51.90, K51.911, K51.912, K51.914, K51.918, K51.919 | |
| Systemic Lupus Erythematosus | 6954, 7100 | M32.0, M32.1, M32.11, M32.12, M32.13, M32.14, M32.15, M32.19, M32.9 | |
| Rheumatoid Arthritis | 7140, 7141, 7142, 7143, 71430, 71431, 71432, 71433 | M05.60, M05.61, M05.62, M05.63, M05.64, M05.65, M05.66, M05.67, M05.69, M05.7A, M05.70, M05.71, M05.72, M05.73, M05.74, M05.75, M05.76, M05.77, M05.79, M05.80, M05.81, M05.82, M05.83, M05.84, M05.85, M05.86, M05.87, M05.89, M05.8A, M05.9, M06.00, M06.01, M06.02, M06.03, M06.04, M06.05, M06.07, M06.08, M06.09, M06.0A, M06.80, M06.81, M06.82, M06.83, M06.84, M06.85, M06.86, M06.87, M06.88, M06.89, M06.9, M08.00, M08.01, M08.02, M08.03, M08.04, M08.05, M08.06, M08.07, M08.08, M08.09, M08.0A. | |
| Down Syndrome | 7580 | Q90.0, Q90.1, Q90.2, Q90.9 | |
| Antibiotic Resistance | V090, V091, V092, V093, V094, V0950, V0951, V096, V0970, V0971, V0980, V0981, V0990, V0991 | Z16.10, Z16.19, Z16.20, Z16.21, Z16.22, Z16.24, Z16.29, Z16.30, Z16.35 | |
